# The association between 24-hour activity, sedentary and sleep compositions and mental health in Australian adults: a cross-sectional study

**DOI:** 10.1186/s44167-023-00024-6

**Published:** 2023-08-01

**Authors:** Rachel G Curtis, Dot Dumuid, Hamish McCabe, Ben Singh, Ty Ferguson, Carol Maher

**Affiliations:** 1 Alliance for Research in Exercise Nutrition and Activity (ARENA), University of South Australia, Adelaide, South Australia Australia; 2 Allied Health and Human Performance, University of South Australia, GPO Box 2471, 5001 Adelaide, SA Australia

**Keywords:** Physical activity, Sedentary behaviour, Sleep, Depression, Anxiety, Stress, Health

## Abstract

**Background:**

Physical activity, sedentary behaviour, and sleep are key components to health and well-being. Compositional data analysis of activity data overcomes the limitations of traditional statistical approaches and comprehensively assesses the association of all activities throughout a 24-hour day. Few studies have used compositional analysis to explore associations between movement behaviours and mental health. This study examined the association between 24-hour activity compositions and mental health in middle-aged Australian adults.

**Methods:**

This study used cross-sectional data from participants (n = 322; mean age 40.4 years; 58.1% female; 47.8% university degree; 84.8% partnered) in the longitudinal *Annual Rhythms in Adults’ lifestyle and health* study (Adelaide, Australia). Activity composition (sleep, sedentary behaviour, light physical activity, and moderate-to-vigorous physical activity) was derived using 24-hour Fitbit accelerometry from the first month of participation (December). Mental health outcomes (depression, anxiety, and stress) were obtained from the Depression Anxiety Stress Scale 21-item short-form (DASS-21). The associations between activity composition (conveyed as isometric log ratios) and DASS-21 scores were examined using compositional multi-level linear regression models with a random intercept for clustering of individuals within families. Using the compositional time reallocation model, expected differences in mental health were examined for hypothetical time reallocations between individual activities.

**Results:**

Favourable associations were observed when time (15 min) was reallocated to light physical activity from sleep (depression: -0.31 [95% CI=-0.57: -0.06]; anxiety: -0.20 [95% CI=-0.37: -0.03] and from sedentary behaviour (depression: -0.29 [95% CI=-0.46: -0.13]; anxiety: -0.14 [95% CI=-0.25: -0.03]; stress: -0.16 [95% CI=-0.31: -0.01]). Detrimental associations were observed when time was reallocated away from light physical activity to sleep (depression: 0.32 [95% CI = 0.07: 0.58]; anxiety: 0.20 [95% CI = 0.03: 0.37]) and to sedentary behaviour (depression: 0.30 [95% CI = 0.13: 0.48); anxiety: 0.15 [95% CI = 0.03: 0.26]; stress: 0.17 [95% CI = 0.01: 0.32]). There were no associations when time was allocated to or from moderate-to-vigorous physical activity.

**Conclusion:**

The way adults spend their time across a 24-hour day is associated with their mental health. Spending more time in light physical activity appears favourable if this time is taken from sleep and sedentary behaviour. These findings provide support for “move more, sit less” public health messages.

**Trial registration:**

This study was prospectively registered on the Australian New Zealand Clinical Trial Registry (Trial ID: ACTRN12619001430123) on the 16/10/2019.

## Background

Mental health disorders are one of the leading causes of the global health-related burden, with substantial costs to individuals and society [1, 2]. One in eight people worldwide were affected by a mental health disorder in 2019 [3], and 44% experience a mental health disorder in their lifetime [4, 5]. Anxiety is the most prevalent mental health disorder [3], and depression is the leading cause of mental health-related disease burden [6]. Furthermore, the worldwide prevalence of stress and distress ranges between 35 and 38% [7–9]. Overall, the annual global costs of mental health disorders is an estimated $2.5 trillion, and this is projected to increase to $6 trillion by 2030 [10].

Physical activity, sedentary behaviour, and sleep each impact the overall health and wellbeing of individuals [11–13]. Higher physical activity and adequate amounts of sleep are consistently associated with improved mental health [14–19], while excessive sedentary behaviour can adversely affect mental health and may increase the risk of future chronic diseases [20–24]. Yet, only 17% of Australian adults meet the national physical activity guidelines [25, 26] and 40%, have difficulty achieving recommended levels of sleep of seven to nine hours a night [27, 28]. Furthermore, 70% of Australian adults spend more than 4 h per day sitting [29], and 40% of their waking time was spent online in 2020 (a 10% increase from 2019 [30]).

To date, most studies have evaluated physical activity, sedentary behaviour, and sleep as independent factors impacting mental health. Yet, these behaviours are not truly independent factors. There are always 24-hours within a day, so if time spent in one activity increases, time in another must decrease [31]. Therefore, evaluating the whole 24-hour day is important to comprehensively understand the association between activity compositions (physical activity, sedentary behaviour, and sleep) and mental health. Until recently, studies generally did not examine the whole 24-hour day since the variables are perfectly multi-collinear [32]. Perfect multi-collinearity indicates perfect linear relationships between multiple variables. The issue of perfect multi-collinearity can be overcome through compositional data analysis (CoDA); a statistical approach which has relatively recently been applied in behavioural research [31]. The use of CoDA with activity data allows a comprehensive assessment of the association of all daily activities and mental health. CoDA expresses the 24-hour (1440-minute) data as a set of isometric log ratios constructed to represent all relative information about the activity data, whilst avoiding perfect multi-collinearity [33]. Interpretation of compositional models is relative; changes in one activity are always accompanied by changes in one or more other activities. Effect sizes can be expressed in terms of time reallocations, such as how much mental health is estimated to differ when time is reallocated from one activity to another within the daily composition.

Few studies have used CoDA to analyse the associations between daily activities and mental health in adults. Findings from these studies have reported that reallocating from time at work to time spent sleeping or undertaking physical activity is associated with improved mental health [33], and that reallocating time from sedentary behaviour and light physical activity to moderate-to-vigorous physical activity or sleep is associated with decreased anxiety [34]. In contrast, other studies have reported no significant associations between changes in activity compositions and mental health [35, 36]. However, limitations of previous work include the use of self-report measures of activity compositions [33] (which may introduce recall or social desirability bias), and the focus on specific populations including retiring older adults [33], adult office workers [35], adults with chronic obstructive pulmonary disease (COPD) [34] and young and middle-aged adults [36]. The way adults spend their time in physical activity, sedentary behaviour, and sleep may have important implications for their mental health, yet further research is required to explore these associations among more representative samples whilst incorporating objective measures of 24-hour activity compositions. Therefore, the aims of this study were to use CoDA to evaluate how objectively measured 24-hour physical activity, sedentary behaviour, and sleep were associated with symptoms of depression, anxiety, and stress among middle-aged adults.

## Methods

### Design

The study used data from a prospective cohort study named “*Annual Rhythms in Adults’ lifestyle and health*” (ARIA), which examined changes in daily activity patterns, diet, weight, and mental health symptoms in adults across a year [37]. The ARIA study was approved by the University of South Australia Human Research Ethics Committee (Protocol number: 201,901). Participants provided informed and written consent before beginning the study. The study was registered on the Australian New Zealand Clinical Trial Registry (Trial ID: ACTRN12619001430123).

### Participants and procedure

The ARIA study consisted of a sample of 375 adults recruited in three waves from greater metropolitan Adelaide, South Australia, who were followed for 13 months. Waves one and two were parents and guardians of children partaking in an ongoing children’s cohort study, named the “*Life on Holidays*” study, recruited in SES strata [38]. Wave three were parents of primary school-aged children recruited from the general population using Facebook posts and advertisements, and through referrals from existing participants. Wave three participants were purposively sampled to address sample imbalances from waves one and two (i.e. wave three emphasised the recruitment of lower SES and male participants). ARIA wave one participants commenced in December 2019, and wave two and three participants commenced in December 2020.

Inclusion criteria were: 18 to 64 years of age, residing in greater metropolitan Adelaide, South Australia, access to a Bluetooth-enabled phone, tablet, desktop computer, or laptop, internet access, parent/guardian of a child involved in the “*Life on Holidays*” study (or parent/guardian of a primary school-aged child for wave three), ambulant, and able to read English. Exclusion criteria were: an implanted electronic medical device, pregnancy, having a life-threatening or serious illness that impacted daily lifestyles.

A baseline face-to-face home visit was conducted prior to the start of data collection. During the home visit, participants were provided with a Fitbit Charge 3, a Fitbit Aria 2 smart body weight scale or a Fitbit Aria Air scale (Fitbit Inc., San Francisco, CA, USA) and a weigh-in reminder flyer. During the home visit, participant’s height was calculated to the nearest 0.1 cm using a stadiometer (Leicester Height Measure MKII, England) following the International Society for the Advancement of Kinanthropometry assessment procedures [39]. In addition, participants completed an online or paper baseline survey regarding their work status, family structure, household characteristics, and demographic information (age, sex, income, and education). Online surveys (delivered using Survey Monkey software) were used to capture diet and mental health at eight time points throughout the year (December, January, March, April, June, August, October, and December). Participants were provided with a $100 honorarium at study completion and were allowed to keep their Fitbit equipment. For the secondary analysis presented here, mental health data were obtained from the January survey (which recalled December) and 24-hour activity data were from the Fitbit data recorded in the first December period (December 2019 for wave one, and December 2020 for waves two and three).

### Variables

**Demographics.** Participants self-reported their date of birth, sex, marital status (never married, widowed, divorced, separated but not divorced, married or de facto), highest education level (year 12 or lower, certificate III/IV, diploma, advanced diploma, bachelor, postgraduate, or higher degree), household gross income (AU$; <$50,000, $50,000-$99,999, $100,000-$199,999, >$200,000), and current smoking status (yes, no).

**Body weight.** Body weight was assessed by the Fitbit Aria smart scales. Bodyweight measures using the Fitbit Aria have shown to be within ≤ 0.5 kg compared with a certified scale in healthy adults [40]. Participants were asked to weigh themselves at the same time each day, preferably in the morning after voiding, wearing minimal clothing, and before eating or drinking. Data were synced to the participants’ Fitbit accounts and were gathered remotely by the purpose-built research software called Fitnesslink, developed by Portal Australia for the ARIA study. The first valid weigh-in (i.e., on, or after 1st December) was used to calculate body mass index.

**Activity compositions.** Daily minutes of sleep, sedentary behaviour, light physical activity, and moderate-to-vigorous physical activity were derived from Fitbit Charge 3 fitness trackers. Participants continuously wore the Fitbit Charge 3 on their non-dominant wrist for 24 h a day over 13 months, including whilst sleeping but excluding when undertaking water-based activities or when charging the tracker. The first month of activity data (December) was used for the analysis. One month of data was chosen to match the survey time frame, which asked about activities and mental health in the last month. Various Fitbit models have shown acceptable reliability and validity for sleep (Fitbit Charge 2 compared to polysomnography 0.61 specificity and 0.96 sensitivity [41, 42]), moderate-to-vigorous physical activity (Fitbit Flex compared to Actigraph GT3X + r = 0.73 [43]), sedentary time (Fitbit Charge 3 compared to activPAL: ICC = 0.94, 95% CI: 0.92–0.96 [44]), and total daily energy expenditure (Fitbit Flex assessed against doubly labelled water in free-living conditions r = 0.33 [45]). Data captured from the Fitbit tracker were synced to the participants’ Fitbit accounts and were gathered remotely via the Fitnesslink software.

Each minute of the day was classified as one of five activities: sleep, sedentary, light, moderate, or vigorous physical activity by Fitbit’s proprietary algorithm. Daily moderate-to-vigorous physical activity was totalled as the number of minutes spent in moderate and vigorous activity. Minutes determined as sedentary (logged when other activities were not identified) and with absent heart rate data were classified as non-wear. Valid days were characterised as a minimum of 18 h wear time including a sleep period. Only participants who had at least three weekdays and one weekend day in the first December of valid data were included in the analysis [46].

**Mental health.** Symptoms of depression, anxiety, and stress were assessed using the DASS-21 [47]. The DASS-21 is a 21-item questionnaire addressing the areas of stress, anxiety, and depression felt over the past month by participants. Participants responded with answers ranging from zero “did not apply to me at all” to three “applied to me very much, or most of the time”. As recommended, to enable comparability with the DASS-42 long version, the DASS-21 raw scores were doubled. The DASS-21 has demonstrated validity and reliability (Cronbach’s alpha 0.82–0.90 for all three subscales [48–50]).

### Statistical analysis

Means and standard deviations were used to represent DASS-21 scores for the pooled sample. The average daily activity composition of sleep, sedentary behaviour, light physical activity, and moderate-to-vigorous physical activity were described using compositional means (geometric means of each activity composition, adjusted linearly so that collectively the behaviours amounted to 1440 min). The associations between activity composition (conveyed as isometric log ratios) and DASS-21 depression, anxiety and stress scores were examined using compositional multi-level linear regression models with a random intercept to account for clustering of individuals within families. Using a cross-sectional compositional time reallocation model conducted in R version 4.1.0 using the compositions package, expected differences in mental health were examined for time reallocations between individual activities [36]. All models were tested for outlying observations, normality, linearity, and homoscedasticity of residuals to ensure assumptions were not violated. Covariates included sex, age, and education. Model-based dose-response curves quantified the expected difference in mental health for time reallocations between activities (e.g., increasing sleep by 20 min by reducing sedentary behaviour by 20 min). Descriptive analyses were carried out in SPSS (V.25, International Business Machines Corporation, New York, NY, USA).

## Results

### Demographics

A total of 322 participants had valid DASS-21 and activity data and were therefore included in the analysis. Participant baseline characteristics are shown in Table 1. Participants comprised of 58.1% females, with an average age of 40.4 years (standard deviation, SD = 5.9 years), were mainly non-smokers (91.9%), and had an average BMI of 28.7 kg/m^2^ (SD = 6.2). Participants were mostly well educated (47.8% bachelor, postgraduate, or higher degree), partnered (84.8% married or de facto), and approximately half had an overall household income of between AU$100,000 and AU$199,999 (47.7%) (Table 1). Participants had an average of 25 valid days of accelerometry data (SD 6, range 6–31) with an average of 23.1 h of wear time (SD 1.3, range 18.0–24.0). On average, participants spent approximately seven and a half hours per day sleeping, ten hours per day sedentary, five hours per day undertaking light physical activity, and half an hour per day undertaking moderate-to-vigorous physical activity at baseline.

**Table 1:** Participant demographic descriptive statistics (n = 322)

Characteristic	Mean (SD) or n (%)	Median (IQR)
Age (y)	40.4 (5.9)	
Sex		
Male	135.0 (41.9%)	
Female	187.0 (58.1%)	
Marital status		
Never	22.0 (6.8%)	
Separated, divorced, widow	27.0 (8.4%)	
Married or defacto	273.0 (84.8%)	
Education level		
Year 12 or lower	59.0 (18.3%)	
Certificate III/IV, diploma, or advanced diploma	109.0 (33.9%)	
Bachelor, postgraduate, or higher degree	154.0 (47.8%)	
Income (AU$)		
<$50,000	32.0 (9.9%)	
$50,000-$99,999	96.0 (29.8%)	
$100,000-$199,999	153.0 (47.5%)	
≥ 200,000	41.0 (12.7%)	
Smoking status		
No	296.0 (91.9%)	
Yes	26.0 (8.1%)	
Body mass index, kg/m^2^	28.7 (6.2)	
MVPA, mins/day	32.7 (22.0)	28.1 (15.6–46.6)
Light PA, mins/day	313.8 (67.6)	313.7 (265.0–359.6)
Sedentary, mins/day	609.0 (79.2)	608.8 (554.3–663.3)
Sleep, mins/day	485.8 (48.7)	481.5 (454.6–514.3)
DASS-21 Depression	5.6 (6.5)	
DASS-21 Anxiety	3.6 (5.0)	
DASS-21 Stress	9.8 (7.3)	

Abbreviations: DASS, depression anxiety stress scale; SB, sedentary behaviour; LPA, light physical activity; MVPA, moderate-to-vigorous physical activity. Note: Activity variables are shown as arithmetic means, adjusted to a total of 1440 min (24-hour day). Adjusted geometric means Sleep = 490.8, SB = 613.2, LPA = 310.5, MVPA = 25.1. Activity composition range Sleep = 312.7–651.7, SB = 416.2–944.3, LPA = 121.4–488.1, MVPA = 0.53–113.8

### Associations between activity compositions and mental health

Estimated differences and 95% confidence intervals in DASS scores associated with reallocations between individual activities are shown in Table 2. Favourable associations were observed when time (15 min) was reallocated to light physical activity from sleep (depression: -0.31 [95% CI=-0.57: -0.06]; anxiety: -0.21 [95% CI=-0.37: -0.03] and from sedentary behaviour (depression: -0.29 [95% CI=-0.46: -0.13]; anxiety: -0.14 [95% CI=-0.25: -0.03]; stress: -0.16 [95% CI=-0.31: -0.01]). Detrimental associations were observed when time was reallocated away from light physical activity to sleep (depression: 0.32 [95% CI = 0.07: 0.58]; anxiety: 0.20 [95% CI = 0.03: 0.37]) and to sedentary behaviour (depression: 0.30 [95% CI = 0.13: 0.48); anxiety: 0.15 [95% CI = 0.03: 0.26]; stress: 0.17 [95% CI = 0.01: 0.32]). Reallocating time to or from moderate-to-vigorous intensity physical activity was not associated with depression, anxiety or stress.

**Table 2:** Estimated differences (and 95% confidence intervals) in DASS-21 scores for 15-minute reallocations between activities (n = 327)

		15 min is *reallocated away from* this behaviour			
		Sleep	SB	LPA	MVPA
	Depression				
	Sleep		0.02 (-0.22: 0.25)	**0.32 (0.07: 0.58)**	0.20 (-0.78: 1.18)
	SB	-0.02 (-0.25: 0.21)		**0.30 (0.13: 0.48)**	0.18 (-0.77: 1.13)
	LPA	**-0.31 (-0.57: -0.06)**	**-0.29 (-0.46: -0.13)**		-0.11 (-1.12: 0.90)
	MVPA	-0.14 (-0.68: 0.39)	-0.13 (-0.62: 0.36)	0.18 (-0.38: 0.74)	
15 min is *reallocated to* this behaviour	Anxiety				
	Sleep		0.05 (-0.10: 0.21)	**0.120 (0.03: 0.37)**	0.20 (-0.45: 0.86)
	SB	-0.06 (-0.21: 0.10)		**0.15 (0.03: 0.26)**	0.15 (-0.48: 0.78)
	LPA	**-0.20 (-0.37: -0.03)**	**-0.14 (-0.25: -0.03)**		0.01 (-0.67: 0.68)
	MVPA	-0.14 (-0.49: 0.21)	-0.080 (-0.41: 0.24)	0.07 (-0.30: 0.44)	
	Stress				
	Sleep		0.06 (-0.15: 0.27)	0.23 (-0.00: 0.46)	0.09 (-0.81: 1.00)
	SB	-0.07 (-0.28: 0.15)		**0.17 (0.01: 0.32)**	0.03 (-0.84: 0.90)
	LPA	-0.23 (-0.46: 0.01)	**-0.16 (-0.31: -0.01)**		-0.13 (-1.06: 0.80)
	MVPA	-0.00 (-0.58: 0.40)	-0.02 (-0.47: 0.43)	0.15 (-0.37: 0.66)	

Abbreviations: LPA: light physical activity; MVPA: moderate-to-vigorous physical activity; SB: sedentary behaviour

Note: DASS and activity compositions were averaged. Estimates are adjusted for age, sex, education level, and clustering within families. Values in bold (*) are significant at an alpha of 0.05

**Fig. 1:**
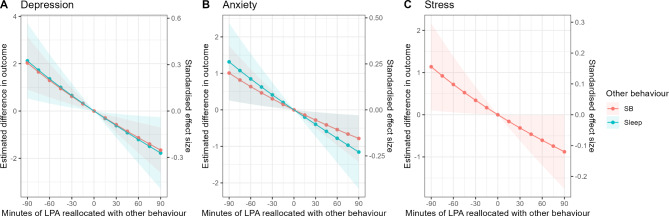
Estimated differences in DASS (**a**) depression, (**b**) anxiety, and (**c**) stress scores (y-axis), associated with reallocations of time spent in light physical activity (x-axis). Note: LPA = light physical activity, SB = sedentary behaviour. Adjusted for age, sex, and highest education. Reallocations between light physical activity and sleep were not significant for stress and are therefore not shown

The expected differences in DASS (a) depression, (b) anxiety, and (c) stress scores for reallocations of time between sleep, light physical activity, and sedentary behaviour, keeping the other activities constant, at the mean composition, is shown in Fig. 1. Reallocations with sleep and sedentary behaviour are shown as separate lines. Negative values on the x-axis indicate time taken away from light physical activity and reallocated to sleep or sedentary behaviour, while positive values on the x-axis indicate time taken away from sleep or sedentary behaviour and reallocated to light physical activity. Reallocations from sleep and sedentary behaviour to light physical activity were associated with reduced depression scores, whereas reallocations from light physical activity to sleep and sedentary behaviour were associated with higher depression scores. The estimated dose-response curves were relatively linear. Reallocations from light physical activity and sedentary behaviour to sleep were associated with increased anxiety and stress scores, whereas reallocations from sleep to light physical activity and sedentary behaviour were associated with reduced anxiety and stress scores. The estimated dose-response curves were relatively linear.

## Discussion

This study evaluated the association between 24-hour activity compositions and mental health in middle-aged adults. Compositional time reallocation analysis suggested that reallocating time to light physical activity from sleep and sedentary behaviour was associated with lower depression, anxiety, and stress. In addition, reallocating time to more sedentary behaviour and more sleep from light physical activity was associated with poorer mental health. Reallocating time to or from moderate-to-vigorous physical activity had no association with depression, anxiety or stress.

The present findings showed that reallocating more time to light physical activity was associated with improved depression, anxiety and stress. These findings are consistent with findings from previous experimental and observational research, which indicate that higher amounts of light, or low intensity, physical activity are associated with improved mental health outcomes in healthy adults [51–53]. Furthermore, our findings are consistent with previous studies using traditional isotemporal substitution analyses [16] and CoDA analyses [33], which found improved mental health outcomes when time spent sleeping was reallocated to light physical activity [16], and when time spent in sedentary behaviour was reallocated to physical activity [33]. These findings provide support for current national activity recommendations or public health messages such as “move more, sit less”. However, in contrast to previous findings [54, 55], our present findings suggested that moderate-to-vigorous physical activity was not associated with improved mental health outcomes. For example, Nakagawa et al. found that reallocating time to moderate-to-vigorous physical activity (assessed using self-report) was associated with improved mental health symptoms, including depression, anxiety, and stress among young adults [54]. Differences in our findings, compared with previous findings may be attributed to the different samples (middle age versus young adults only) and different methods to assess physical activity (objective versus self-report).

In particular, we collected activity data using Fitbit fitness trackers, and the findings showed that, on average, participants undertook approximately half an hour of moderate-to-vigorous physical activity per day. Previous studies have used fitness trackers or accelerometers from a variety of manufacturers to measure activity compositions. In addition, varying cut points have been used to determine time spent in energy expenditure bands, resulting in different classifications. For example, in a study looking at participants with COPD, participants undertook approximately 90 min of moderate-to-vigorous physical activity a day [34]. It appears that the Fitbit algorithms may have a higher cut point for defining activity therefore underestimating moderate-to-vigorous intensity compared to research accelerometers [56]. This may explain why significant associations were detected for light physical activity but not moderate-to-vigorous intensity physical activity in our study.

Our findings showed that reallocating more time towards sleep was associated with worse mental health. This finding may be considered unexpected, and there is a general understanding that more sleep is beneficial [12, 57]. For example, Olds et al. found that older adults in retirement slept for seven to nine hours a night on average and increased sleep durations were associated with improved mental health [33]. However, previous studies have found that sleeping for greater than nine hours a night is associated with poorer mental health outcomes [57–59]. On average, participants in the present study slept for approximately seven and a half hours a night, which sits within recommended guidelines [28]. It is possible that there is an optimal sleep duration, beyond which the mental health associations become detrimental (i.e., an inverted U-shaped relationship). Further research among participants that are sleep deprived may produce different results. Alternatively, given the cross-sectional nature of the analysis, the results may be explained by reverse causation, whereby having poorer mental health leads to longer sleep durations.

This study has a number of strengths. It is one of the first studies to analyse the association between activity compositions and mental health using CoDA, thus contributing to the growing evidence in this area. The activity data were collected using activity trackers, therefore minimising the risk of recall and selection bias [33, 34]. This study used a 24-hour wear protocol with a threshold of 18 h to define a valid day and 4 days of data, including a weekend day, for inclusion in the analysis. This resulted in a large volume of data, with included participants having an average of 25 valid days of data with 23 h of data per day. We note, however, that there could still be systematic errors given that wear time may be less complete during certain times of the day or on certain days.

Limitations of this work include the cross-sectional design, therefore limiting the ability to infer causality of associations. Fitbits may use different cut points to classify activities than research accelerometers, making comparison of results more difficult. Participants were residents of the greater Adelaide region and had relatively high educational attainment and income, therefore the generalisability of these findings to other populations is unclear. Furthermore, the sample’s stress, anxiety, and depression scores were generally within normal ranges. It is likely that results may be different among samples with worse mental health outcomes. We note that data for wave 2 and 3 participants were collected during the COVID-19 pandemic. At this time, there were some continuing restrictions in Adelaide, Australia, including international travel restrictions and density restrictions in public venues, which may have affected participants’ activity and mental health, though participants’ daily lives were relatively unrestricted compared to most countries.

Future research evaluating the longitudinal associations between daily activity compositions and mental health would provide further information about the potential direction of the observed relationships. Additionally, research in different population, such as adults with more symptoms of depression, anxiety and stress or diagnosed mental health disorders, is needed.

In conclusion, this study examined how activity compositions were associated with mental health in a sample of middle-aged adults from Adelaide, Australia. The 24-hour activity compositions (sleep, sedentary behaviour, light physical activity, moderate-to-vigorous physical activity) were associated with symptoms of depression, anxiety, and stress. Reallocation of time to light physical activity from sleep or sedentary behaviour was associated with the most beneficial associations with mental health. Findings suggest that among healthy adults, encouraging more light physical activity may help optimise mental health.

## Data Availability

The datasets analysed during the current study are available from the corresponding author on reasonable request.

## Abbreviations

ARENA: Alliance for Research in Exercise, Nutrition and Activity
ARIA: Annual Rhythms in Adults’ lifestyle and health
CoDA: Compositional data analysis
COPD: Chronic obstructive pulmonary disease
DASS: Depression Anxiety Stress Scale
HREC: Human Research Ethics Committee
NHMRC: National Health and Medical Research Council
